# Functional morphological study on the tongue of the adult Egyptian geese (Alopochen egyptiacus)

**DOI:** 10.1186/s12917-025-04745-5

**Published:** 2025-05-07

**Authors:** Fatma Abdelhakeem, Salma A. Mohamed, Mohammed Abdelsabour-Khalaf, Soha Soliman, Kamal E. H. Abdalla

**Affiliations:** 1 Department of Anatomy and Embryology, Faculty of Veterinary Medicine, South Valley University, Qena, 83523 Egypt; 2 Department of Histology, Faculty of Veterinary Medicine, South Valley University, Qena, 83523 Egypt; 3 Department of Anatomy and Histology, Faculty of Veterinary Medicine, Assiut University, Assiut, Egypt

**Keywords:** Geese, Tongue, Stratified epithelium, Salivary glands, Lingual papillae

## Abstract

**Background:**

The present investigation represents the first attempt to fully describe the morphology of the geese tongue. Tongue has an essential biological role in all vertebrates, it is considered a prehensive organ, as it is responsible for food collection, also aids in mastication and swallowing. Since the tongue morphology was varied among bird species linked to different reasons such as beak shape, environment, feeding habits and type of diet, that’s why the purpose of this study was to investigate the morphology of the geese tongue in detail concerning its function.

**Methodology:**

Eleven adult healthy Egyptian geese of both sexes were used in this study, the heads of these birds were collected, and the tongue processed for gross investigation, morphometrical analysis, light microscopy, and scanning electron microscopy.

**Result:**

The morphology of geese's tongue was unique, had narrow elongated shape with round thin free apex, with ventral keratinized lingual nail and caudal torus linguae. The apex and body had two groups of different shaped and sized marginal lingual papillae. The rostral group was 9-10 narrow spaced small thin conical papillae with pointed apices on the lateral margin of the tongue apex. And the caudal group was 6-7 widely spaced large thorn like conical papillae, positioned on the lateral margin of the caudal portion of the lingual apex and the body. Geese's tongue supported with entoglossal bone that extended from apex tip to transverse row of papillae where articulate with basibranchial bone.

**Conclusion:**

The collected data described that the geese tongue was elongated in shape, its dorsum covered with keratinized stratified squamous epithelium and had two groups of mechanical marginal papillae which were necessary for the herbivorous birds as geese, depended on grassing as feeding behavior. Besides keratinization is a sign of tongue modification protecting lingual tissue against hard food particles. Conducting morphological results about geese tongue offers guidance and insights for its adaptation function and the management of this bird species.

## Introduction

Tongue is a complex multifunctional organ, that helps in mastication, swallowing, vocalization, thermoregulation, and taste reception [1]. The tongue shape varies among different bird’s species, in domestic goose embryos appears having rounded apex between 10th and 16th day of incubation, changes into spatula-like shape on the 17th day of incubation and converts to triangular shape with rounded tip on the 19th day of incubation [2], but in adult goose appears narrow elongated with rounded apex and ventral keratinized lingual nail which important for pecking the solid grains [3], short and triangular in pigeon, long lanceolate with narrow tapering apex in cattle egret [4] and triangular with a bifurcated tip in the barn owl [5]. The morphological features of the tongue differs depending on anatomy of bird’s beak, feeding habits and living environment [5, 6].

Morphologically, the tongue consists of three main parts; apex, body and root [3, 7, 8], goose has a special structure rostrally, the keratinized lingual nail which is an exoskeleton for the tongue [3], the dorsal lingual surface is characterized with presence dorsal median groove, it has significant function which is to path and direct the food [9] and the entoglossal bone that is a supportive core for goose tongue [3]. The margins of the tongue is characterized with presence of small and giant conical papillae situated in between them filiform papillae, these marginal mechanical papillae are linked functionally with geese feeding mechanism as grazing and filter-feeding [3, 10, 11].

Microscopically, it composed of mucosa, submucosa, and supported core [12]. The mucosa is modified on the dorsum and both sides of tongue forming lingual papillae which some of them have gustatory function and characterize with presence of taste buds as fungiform and vallate papillae, the other type of papillae are mechanical type have role in food cutting, transportation and prevent its regurgitation, sometimes are cornified as filiform, lentiform, coniform and conical papillae [3, 8, 13–15].

The Egyptian geese, or *Alopochen aegyptiaca* is a waterfowl bird that belongs to the Tadorninae subfamily and the family Anatidae [16]. The goose is a popular poultry breed that is introduced as an ornamental species [17], and over the past 20 years, the geese industry has grown significantly globally [18].

Practitioners concerned with geese feeding behavior and their affected diseases require anatomical knowledge to understand the physiology and pathology of this bird. Since the tongue is very important tool for food intake and its morphology varies in relation to habitat and environment [19]. The morphological details about the geese tongue are few such as [2, 3, 10] which discussed the tongue shape and its lingual papillae, so the present investigation was conducted to give a detailed information about the macroscopical, microscopical and morphometrical features of the Egyptian geese tongue using light and scanning electron microscope which aid in understanding its microstructure in relation to its adapted function.

## Materials and methods

### Sample collection and Preparation

The current study was conducted at the Faculty of Veterinary Medicine, South Valley University, Qena, Egypt, following the institutional and national guidelines and regulations for the use and care of birds and according to Egyptian animal law.

Eleven adult healthy Egyptian geese (Alopochena egyptiacus) of both sexes (six males and five females) were used in this study, the birds were collected and obtained from different local markets and farms at local Egyptian village of Qena. According to Madkour and Abdelsabour-Khalaf [20], all birds were anesthetized using a mixture of ketamine and xylazine (1:1) (0.0044 cc/kg) injected into the pectoral muscle, then sacrified and allowed to fully bleed. The heads were cut off after complete bleeding, washed several times with saline solution then fixed in the appropriate solution. Five birds were fixed for 10% buffered formalin for gross morphology, three birds were fixed in 2.5% glutaraldehyde solution for scanning electron microscope and the last three birds were fixed in 10% neutral buffered formalin or Bouin’s solution for light microscopy adopted according to [21, 22].

### Gross and morphometrical examination

The oropharynx opened from the angle of the mouth; its floor was dissected and fixed in 10% formalin, the tongue was carefully dissected from the surrounding structures, after that photographed for the description of Its different morphological features.

The different measurements (length, width and thickness) of the tongue and its parts apex, body and root were recorded using Precision Digital Vernier Caliper, the mean of these measurements was statistically analyzed using IBM SPSS Software (version 22) and was expressed in millimeters as Mean ± SD.

The anatomical terminology (nomenclature) used in this study was adopted according Nomina Anatomica Avium [23] whenever possible.

### Scanning electron microscopical examination

The analysis of tongue samples had occurred in electron microscopy unit in the faculty of veterinary medicine, Assiut University, Assiut, Egypt.

The tongue of the three birds were washed several times with normal saline and acetic acid 2%, fixed in 2.5% glutaraldehyde solution (pH 7.4) for at least 24 h, then post fixed in 1% buffered osmium tetroxide. The samples were washed in phosphate buffer (pH 7.4), then dehydrated in ascending grades of ethyl alcohol followed by critical point-dried in liquid carbon dioxide. All specimens were mounted on aluminum stubs covered with carbon tabs and sputtered with gold. Specimens were examined and photographed using JEOL scanning electron microscopy (JSM-5400) with accelerating voltage (15kv) according to [24].

### Preparation of tongue for light microscopy

Cross and longitudinal sections from tongue (apex, body, lingual prominence and root), were taken from the collected heads just after scarifying, washed and fixed in 10% neutral buffered formalin or Bouin’s solution for at least 24 h. After that, the samples were kept in decalcifying agent (formic acid solution) to ensure of the decalcification of the bony and cartilaginous contents of tongue (entoglossal bone and hyobranchial apparatus), then tested physically under finger touch, usually the older ages required up to one and half month in formic acid solution. After proper decalcification, the specimens were subjected to dehydration in a series of increasing concentrations of ethanol (70%, 80%, 9o% and100%) for at least three minutes for each, then cleared in methyl benzoate and embedded in paraffin wax [25].

Paraffin blocks were sectioned using a LEICA 2165 microtome with a thickness of 3–5 μm. the sections deparaffinized and stained with several stains, **Harris’s Hematoxylin and Eosin (H&E)** for general histological examination, **Crossmon’s Trichrome**,** Masson’s Trichrome** for demonstration of collagen fibers, **Periodic Acid - Schiff’s reagent and Alcian blue** for observation the neutral and acidic mucopolysaccharide distribution and **Safranin-O** for detection of GAG rich matrix of cartilage. All stain techniques were adopted after [26].

Then examined and photographed microscopically through using DMLS light microscope (Leica, Germany) equipped with an MC120 HD camera.

## Result

### Gross morphology and morphometrical analysis of the tongue

**Fig. 1:**
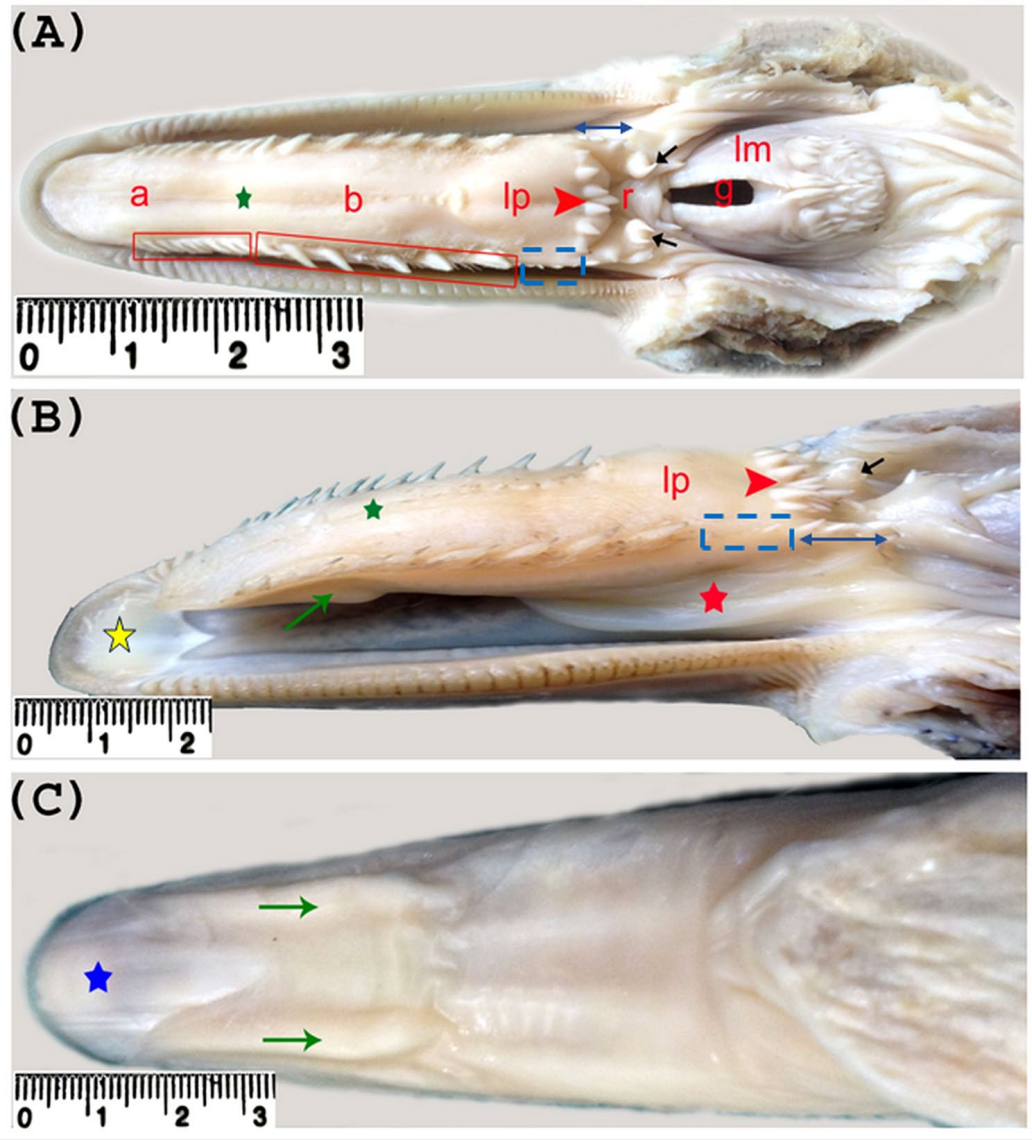
Photographs (**A**-**C**) showing the different morphological structures of the geese’s tongue. Note, apex (**a**), body (**b**), lingual prominence (lp), transverse row of lingual papillae (red arrow head), root (r), two mucosal elevations of the root (black arrow), the rostral group of papillae (small red quadrangular), caudal group of papillae (large red quadrangular), 3–4 papillae representing the caudal continuation of the caudal group papillae ( dotted blue quadrangular), median lingual groove (green star), lateral sided papillae of the root (blue two sided arrow), ventral mucosal projections (green arrow), lingual nail (blue star), area of oral floor free of tongue (yellow star) and the frenulum linguae (red star)

The geese’s tongue, or Linguae, was narrow elongated with round relatively thin free tip. It did not fill the oral cavity floor completely and there was an area free from the tongue extended from its free tip to the free tip of the lower beak, measured 6.67 ± 0.26 mm long (Table 1; Fig. 1B). The tongue was divided into three parts: apex (apex linguae), body (corpus linguae) and root (Radix linguae). The apex was separated from the body at the level of the lingual frenulum that attached the ventral aspect of the tongue into the oral floor, characterized with presence of ventral keratinized structure called lingual nail. A transverse row of caudally directed lingual papillae (papillae linguales) was observed Between the body and root of the tongue (Fig. 1).

**Table 1:** Different measurements and ratios of the Geese’s tongue

Measurements	Mean ± SD
**Total length of tongue (mm)**:	58.10±0.43
-length of apex (mm).	29.14±1.11
-length of body (mm).	19.78±0.76
-length of root (mm**)**.	9.85±0.46
**Ratio (%) of length of**:	
-apex to total length of tongue.	50.04
-body to total length of tongue.	34.04
-root to total length of tongue.	16.94±
**Width (mm) of tongue at**:	
-rostral end of marginal papillae.	9.36±0.30
-in front frenulum linguae.	12.43±0.16
-lingual prominence.	12.69±0.10
-transverse row of papillae.	13.25±0.17
-middle of root.	12.49±0.28
**Thickness (mm) of tongue at**:	
-rostral end of marginal papillae.	4.05±0.17
-in front frenulum linguae.	8.76±0.25
**Median lingual groove**:	
**-**its length (mm).	48.22±0.18
-number of papillae bordering it.	8–10
-length of this row of papillae (mm).	12.70±0.26
**Lingual prominence dimensions (mm)**:	
-length.	7.65±0.15
-width at its base.	6.37±0.18
-its position rostral to transverse row of papillae.	5.61±0.20
**Length of oral floor free from tongue (mm).**	6.67±0.26

The statistical analysis revealed that the total length of the tongue was 58.10 ± 0.43 mm, while that of the apex, body and root was 29.14 ± 1.11, 19.78 ± 0.76 and 9.85 ± 0.46 mm respectively. The ratio of the length of these parts to the total lingual length was about 50% for the apex, 34% for the body and 16% for the root, indicated that the apex represented the rostral half of the total lingual length, however, the body and the root constituted its caudal half. Additionally, the length of the apex of the tongue formed nearly one and half folds that of the body and three folds that of the root (Table 1).

The tongue had nearly a constant width ranging from 12.43 ± 0.16 to 13.25 ± 0.17 mm, but it was relatively narrow and thin at its free tip measuring 9.36 ± 0.30 mm in width and 4.05 ± 0.17 mm in thickness (Table 1; Fig. 1). With regards to the thickness, the lingual apex was thin rostrally measuring 4.05 ± 0.17 mm and increased in thickness two folds to become 8.76 ± 0.25 mm in front of the frenulum linguae (Table 1).

Two nearly triangular shaped lingual prominences (torus linguae) were demonstrated caudally on the dorsal surface of the lingual body, about 5.61 ± 0.20 mm rostral to the transverse row of the lingual papillae. They bordered both sides of the caudal part of the median lingual sulcus. The base of each prominence was directed caudally. The lingual prominence measured 7.65 ± 0.15 mm long and 6.37 ± 0.18 mm wide at its base (Table 1; Fig. 1).

The dorsal surface of the apex and body of the tongue was characterized with presence of median longitudinal groove (sulcus lingualis medianus) which began shortly behind the tip of the tongue, extended caudally to terminate at the transverse row of the caudally directed lingual papillae, measured 48.22 ± 0.18 mm long. Rostral to the lingual prominence, the median groove was bordered linearly on both sides for about 12.70 ± 0.26 mm by 8–10 small papillae (Table 1; Fig. 1).

Two elongated ventrolateral mucosal projections were demonstrated ventrally on the rostral free part of the tongue. They began behind the tip of the tongue and extended for about 13.18 ± 0.28 mm caudally, measured 7.43 ± 0.19 mm wide at the middle, they increased in width caudal wards (Table 1; Fig. 1B, C).

The transverse row of lingual papillae which separated the body from the root of the tongue lied opposite to the angle of the mouth and joined laterally the papillae on the lateral edges of the tongue. It contained 7–9 papillae and measured about 10.96 ± 0.31 mm long. The transverse row of the lingual papillae showed a marked convexity directing caudally at its middle (Table 2; Fig. 1A, B).

**Table 2:** The marginal lingual papillae

Measurements	Mean ± SD
Rostral group (row):	
**-number of papillae.**	9–10
**- Length of the row (mm).**	12.43±0.61
**-beginning caudal to lingual tip (mm).**	7.69±0.49
**-end in front frenulum linguae (mm).**	12.61±0.43
**Caudal group (row)**:	
**-number of papillae.**	6–7
**-length of the row (mm).**	20.57±0.62
**-beginning rostral to frenulum linguae (mm).**	10.53±0.09
**-end in front transverse row of lingual papillae (mm).**	9.18±0.18
**Transverse row of lingual papillae**:	
**-number of papillae.**	7–9
**-length of this row (mm).**	10.96±0.31

A short row of 5–6 short caudolaterally directed papillae extended caudally from each end of the transverse row of the lingual papillae, formed the lateral boundary of rostral part of the root of the tongue. These papillae increased in length caudal wards and were mainly conical in shape, but the most caudally placed ones had abroad roots (Fig. 1A, B).

The root of the tongue extended from the transverse row of the lingual papillae to the laryngeal mound, occupying the rostral part of the pharyngeal floor. Its rostral part situated immediately caudal to the transverse row of the lingual papillae was deeper and grossly smooth than its caudal part which terminated at the rostral commissure of the laryngeal inlet having several fine papillae. The root of the tongue was wider rostrally than caudally and bounded laterally as mentioned before by short row of 5–6 caudolaterally directed papillae. Caudal to each row of papillae, the root of the tongue was marked laterally by a mucosal elevation containing 5–6 different sized cone shaped caudally directed papillae (Fig. 1A & B).

The lateral margin (margo linguae) of the apex and body of the tongue was characterized by presence of different shaped and sized marginal lingual papillae which directed caudolaterally and slightly dorsally, they divided into two groups: rostral and caudal. The rostral group was 9–10 papillae, measured 12.43 ± 0.61 mm long, situated on the lateral margin of the tongue apex and grossly they were narrow spaced small thin conical papillae with pointed apices. This rostral group started 7.69 ± 0.49 mm behind the lingual free tip and ended 12.61 ± 0.43 mm in front of the lingual frenulum. The caudal group was 6–7 widely spaced large thorn like conical papillae, measured 20.57 ± 0.62 mm long, positioned on the lateral margin of the caudal portion of the lingual apex and the body, began in front of the lingual frenulum by about 10.53 ± 0.48 mm and terminated rostral to the transverse row of the lingual papillae by about 9.18 ± 0.18 mm (Table 2; Fig. 1). These caudal papillae increased in size caudal wards and the spaces between them were generally equal except the space between the last one and that before it was larger than the other spaces. There were 3–4 small caudolaterally directed papillae representing the caudal continuation of the caudal group papillae that continued caudally with the short row of papillae which bounded laterally the rostral part of the root of the tongue (Fig. 1A & B).

### Results of scanning electron microscope

**Fig. 2:**
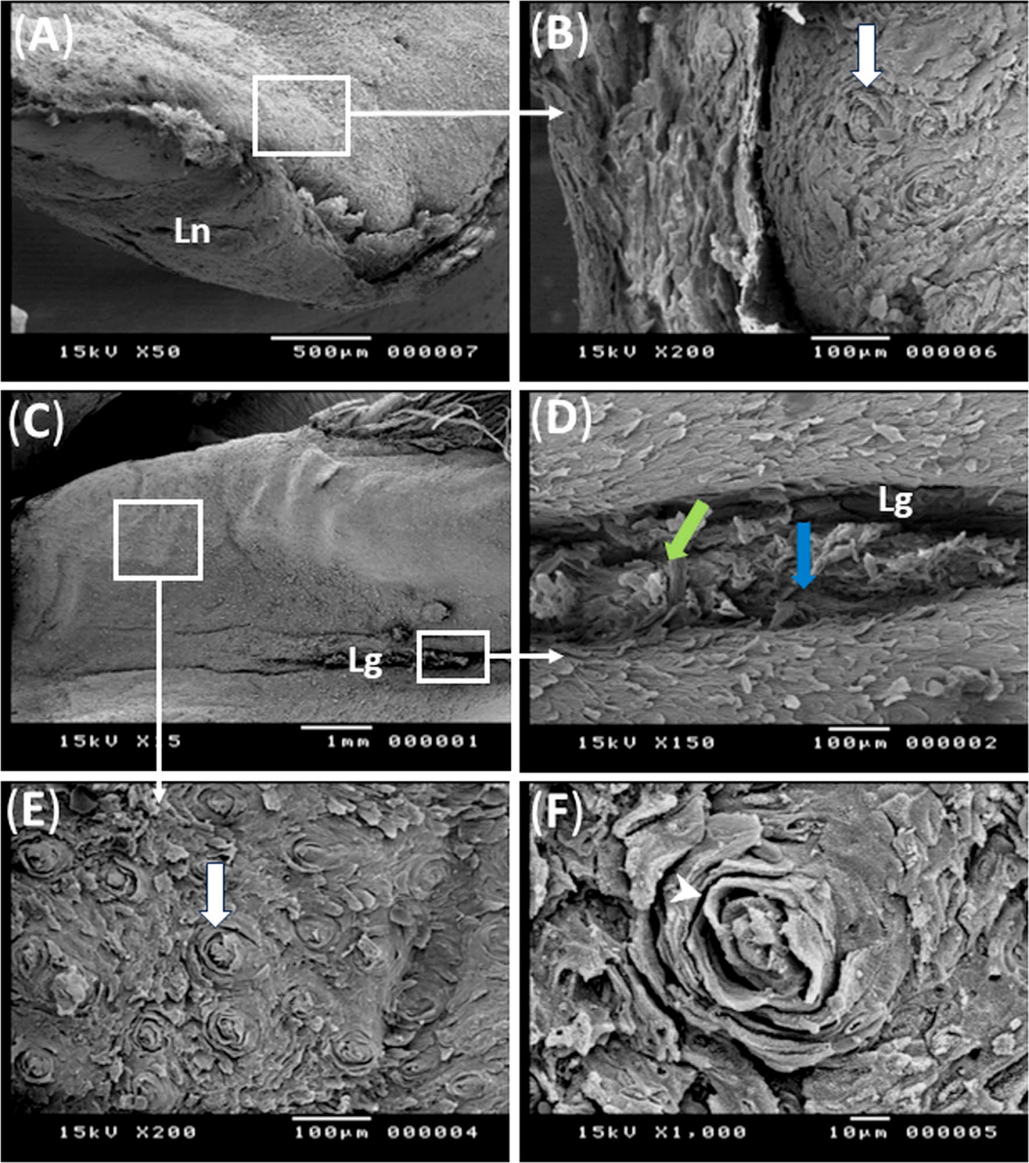
Scanning electron micrographs (**A**-**F**) showing structure of the lingual nail and the dorsum of the lingual apex and body of the geese’s tongue. Note, lingual nail (Ln), median lingual groove (blue arrow and Lg), desquamated cells (green arrow), rosette shaped structures (white arrow), and layers of keratin (white arrow head)

The scanning electron micrograph of the free tip of tongue had thick multilayered keratinized epithelium covering the ventral surface of the tip forming the lingual nail. It protruded from the ventral surface surrounding the rostral and lateral borders of the free tip of the tongue (Fig. 2A).

The scanning electron micrograph of the dorsum of tongue showed that, the free tip of the tongue had many different sized rosette shaped structures which were absent laterally, they were encircled by concentric layers of keratin. These rosette structures decreased in number caudal wards (Fig. 2B, E, F). The median lingual groove appeared as median longitudinal sulcus extends over the dorsum of the lingual apex and body, and contained desquamated cells (Figs. 2C and D and 4A). The cross section of tongue showed its core which formed of entoglossal bone surrounded with dorsal and ventral epithelium (Fig. 4A, B).

**Fig. 3:**
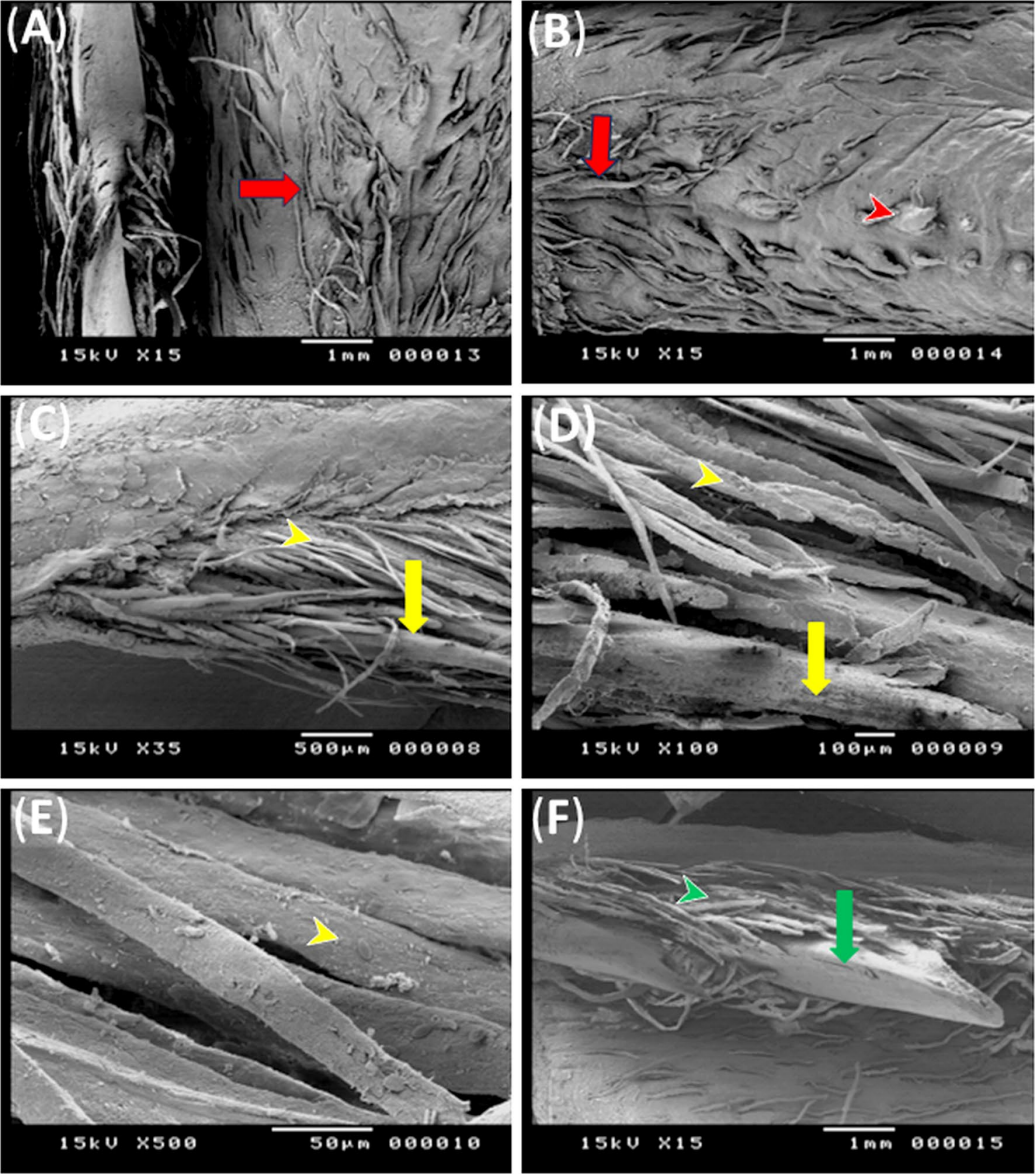
Scanning electron micrographs (**A**-**F**) showing the dorsum papillae, rostral and caudal groups of marginal lingual papillae of the geese’s tongue. Note, filiform papillae (red arrow), broad papillae (red arrow head), cylindrical papillae of rostral group (yellow arrow), haired papillae of rostral group (yellow arrow head), conical papillae of caudal group (green arrow) and filiform papillae of caudal group (green arrow head)

Numerous caudally directed filiform papillae covered the dorsal and lateral aspects of the body of the tongue (Fig. 3A, B). Small few short caudally directed broad papillae were observed in the mid region of the dorsum of the body of the tongue (Fig. 3B).

**Fig. 4:**
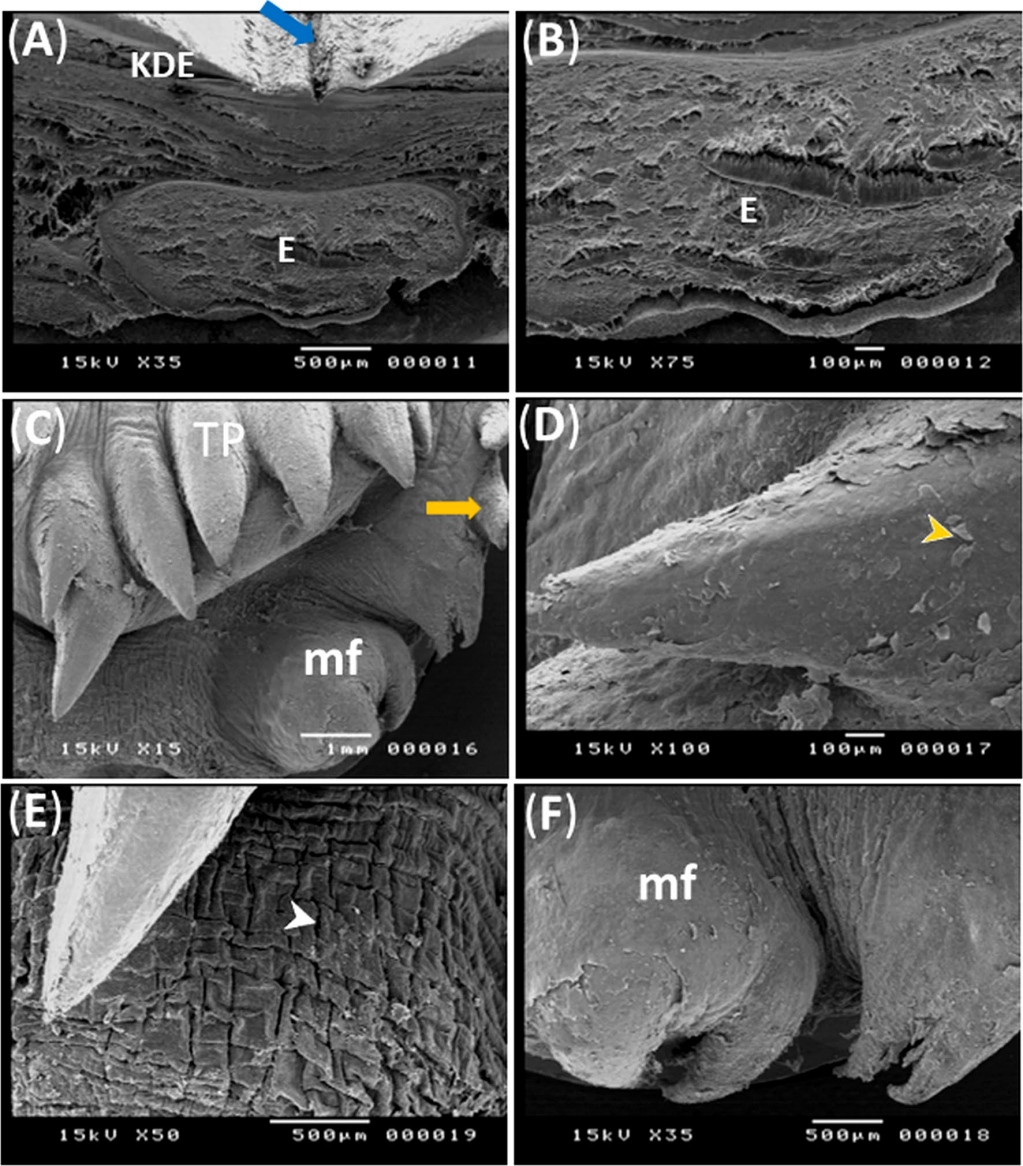
Scanning electron micrographs (**A**-**F**) showing entoglossal bone and structure of the lingual root of the geese’s tongue. Note, the entoglossal bone (**E**), parakeratinized dorsal epithelium (KDE), median lingual groove (blue arrow), conical papillae of transverse row (TP), exfoliated cells (orange arrow head), mucosal elevation (mf), papillae bordering lingual root (orange arrow) and longitudinal grooves (white arrow head)

The rostral group of the marginal papillae were distinguished as two types: long, thick elongated papillae and long thin haired shaped papillae. At higher magnification the former papillae appeared cylindrical in shape and were surrounded dorsally and ventrally by the later papillae, some of these cylindrical papillae were branched. Moreover, the haired shaped papillae appeared flattened (Fig. 3C: E).

The caudal group was conical papillae surrounded with haired shaped caudolaterally filiform papillae which arranged into longitudinal rows dorsal and ventral to large conical papillae, the as dorsally situated ones were mainly long and straight where the ventrally situated ones were relatively short and twisted (Fig. 3F).

The lingual papillae of the transverse row which was situated between the body and root were conical in shape, directed caudally, decreased in size lateral-wards and some of them had broad roots (Fig. 4C). The lingual papillae showed exfoliated cells which began from the roots and extended towards the apices (Fig. 4D). The root of the tongue contained longitudinal narrow grooves (Fig. 4E). The papillae of the mucosal elevations and those bordering the root laterally were wedged shaped (Fig. 4C, F).

### Light microscopical findings

**Fig. 5:**
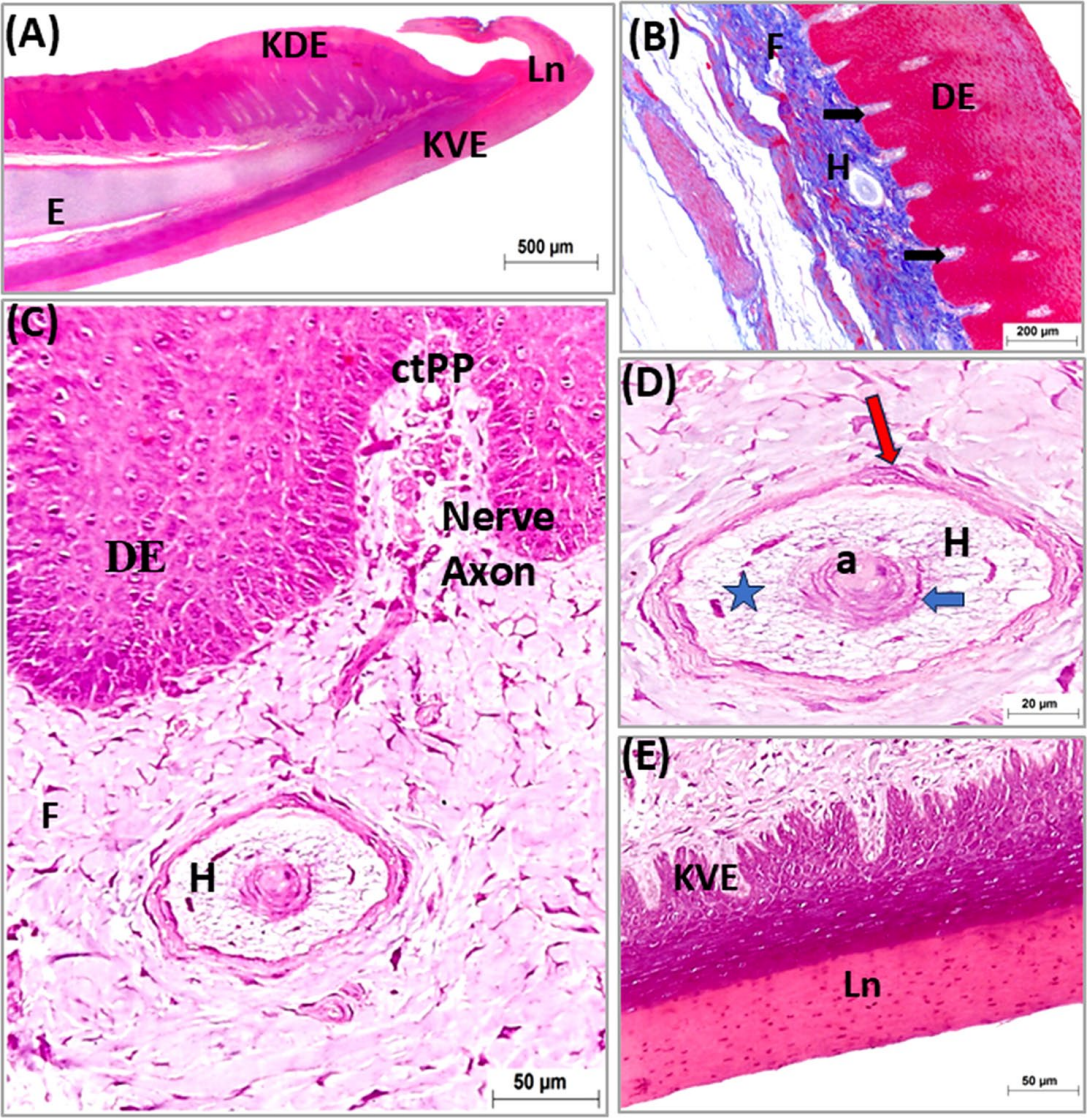
Light micrographs of longitudinal paraffin sections (**A**-**E**) showing structure of the apex of the geese’s tongue. Photos (**A**, **C**-**E D**) stained with H&E and (**B**) with Masson’s Trichrome. Note, the entoglossal bone (**E**), parakeratinized dorsal epithelium (KDE), non-keratinized dorsal epithelium (DE), parakeratinized ventral epithelium (KVE), collagenous connective tissue (f), lingual nail (Ln), Connective tissue papillae (black arrow or ctPP), Herbst corpuscles (**H**), central axon (**a**), internal capsule (blue arrow), collagenous fiber of corpuscle (star) and external capsule (red arrow)

**Fig. 6:**
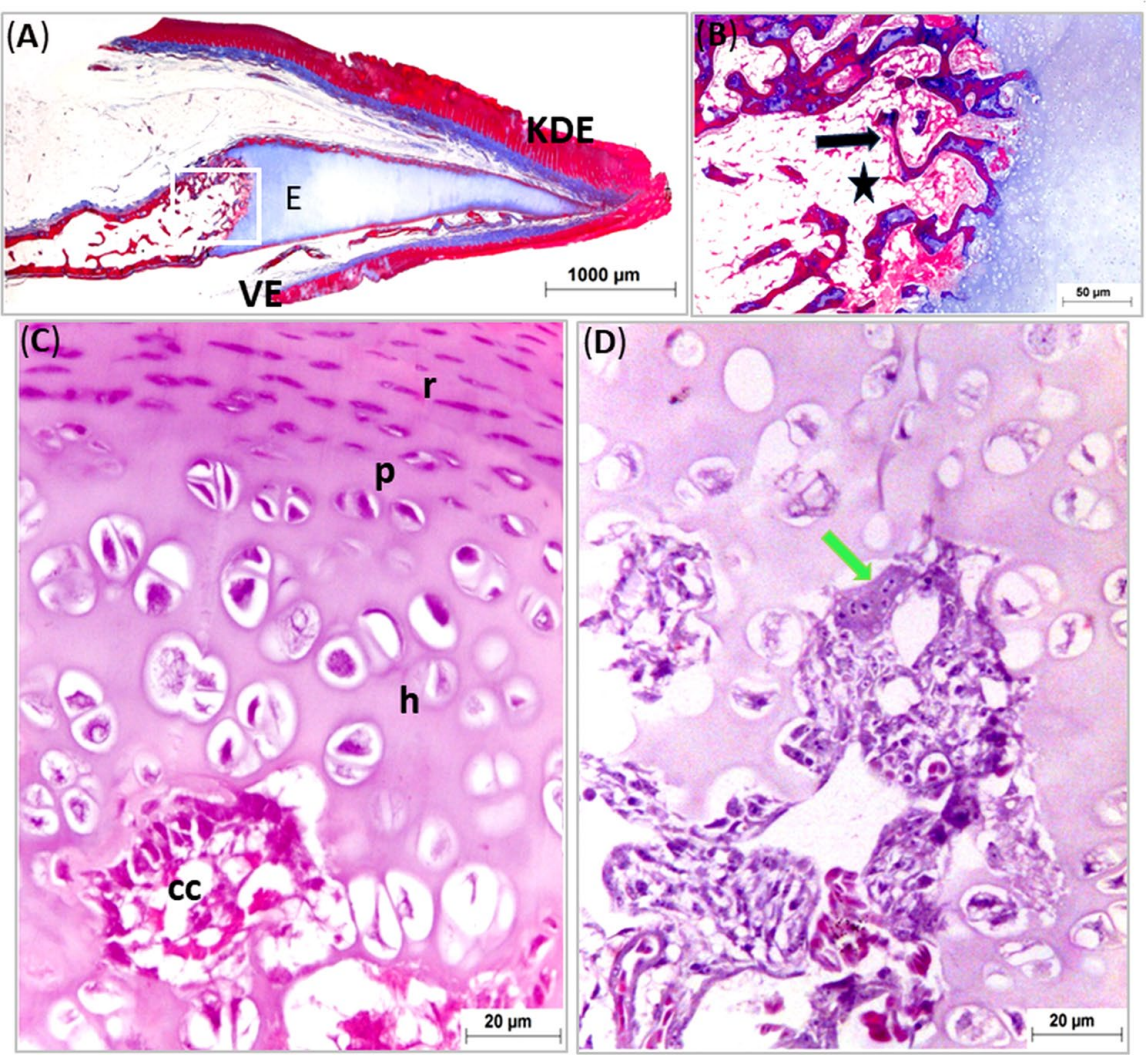
Light micrographs of longitudinal paraffin sections (**A**-**D**) showing ossification of the entoglossum of the geese’s tongue. Photos (**A** & **B**) stained with Masson’s Trichrome (**C** & **D**) with H&E. Note, parakeratinized dorsal epithelium (KDE), ventral epithelium (VE), the entoglossum (**E**), bone trabeculae (black arrow), adipose tissue (black star), cartilaginous canal (cc), osteoclast cell (green arrow), resting zone (r), proliferating zone (P) and hypertrophic zones (h)

The tongue of the geese covered with stratified squamous epithelium at both dorsal and ventral surfaces. The epithelium was generally thicker at the dorsal surface than at the ventral one. The dorsal epithelium was highly thick at the free part, lingual prominence (torus linguae) and marginal papillae (Figs. 5A, 6A and 8A: C).

**Fig. 7:**
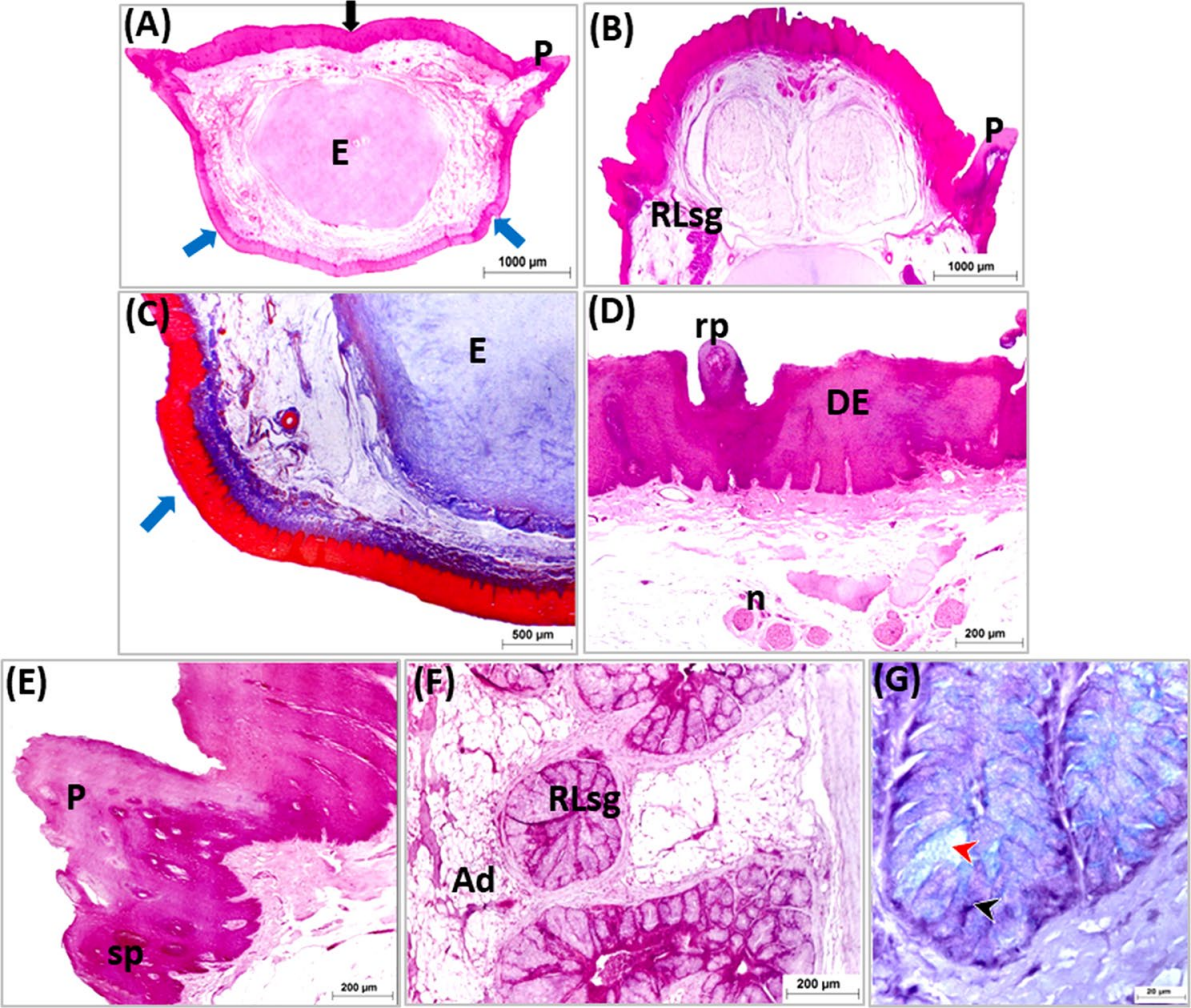
Light micrographs of cross paraffin sections (**A**-**G**) showing structure of the apex at level of two ventrolateral mucosal projections (**A** & **C**) and rostral part of lingual body (B & D-G). Photos (**A**, **B** & **D**-**F**) stained with H&E, (**C**) with Masson’s Trichrome and (G) with PAS/Alcian blue. Note, dorsal epithelium (DE), entoglossum (**E**), two mucosal projections (blue arrow), median groove (black arrow), nerve endings (n), rounded papillae (rp), marginal lingual papillae (P), secondary small papillae (sp), rostral lingual s.g (RLsg), adipose tissue (Ad), mucous cells positive for PAS (black arrow heads) and mucous cells positive for Alcian blue (red arrow head)

**Fig. 8:**
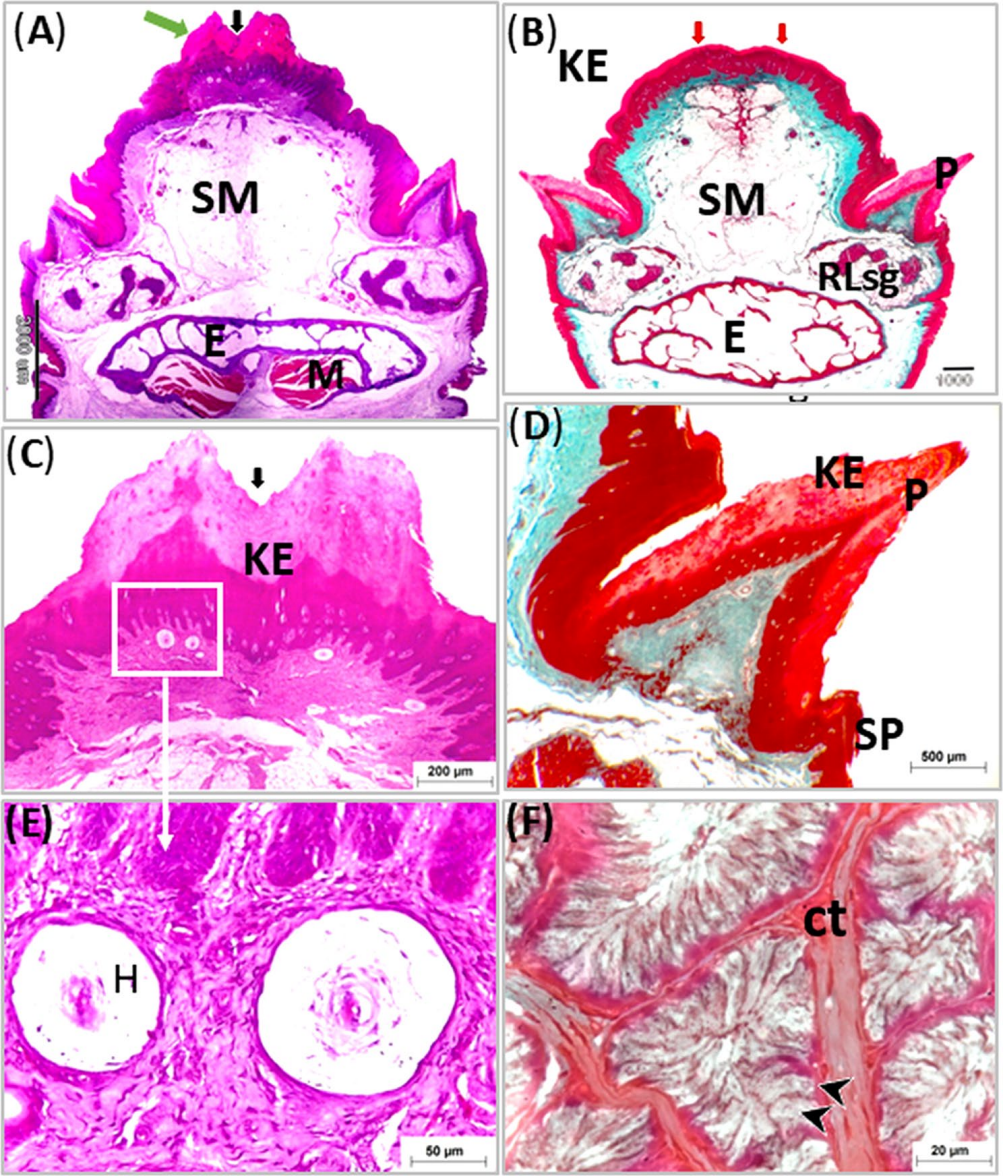
Light micrographs of cross paraffin sections (**A**-**F**) showing structure of the lingual prominence of the geese’s tongue (**B**, **D** & **F**) and area in front it (**A**, **C** & **E**). Photos (**A**, **C** & **E**) stained with H&E and (**B**, **D** & **F**) with Crossmon’s Trichrome. Note, lingual prominences (two red arrow), Keratinized epithelium (KP), submucosa (SM), entoglossum (**E**), median groove (black arrow), dorsal keratinized papillae rostral to lingual prominence (green arrow), marginal papillae (P), small papillae (sp) Herbst corpuscle (**H**), compound rostral lingual glands (RLsg), mucous cells (black arrow heads) and connective tissue septa between lobule of salivary gland

The epithelium on the ventral surface and dorsal surface of the rostral free part of the tongue, and the dorsal epithelium of the lingual prominence appeared para keratinized. The para-keratinization was also detected at lingual papillae (Figs. 5A, 6A and 8A: C). While the other parts of the tongue were covered by non-keratinized stratified epithelium (Fig. 7A: E). The thickened para-keratinized epithelium of the ventral surface of apex formed the lingual nail (Fig. 5A, E).

The ventral mucosa of the lingual apex caudal to free tip of tongue formed two mucosal projections (Fig. 7A, C).

**Fig. 9:**
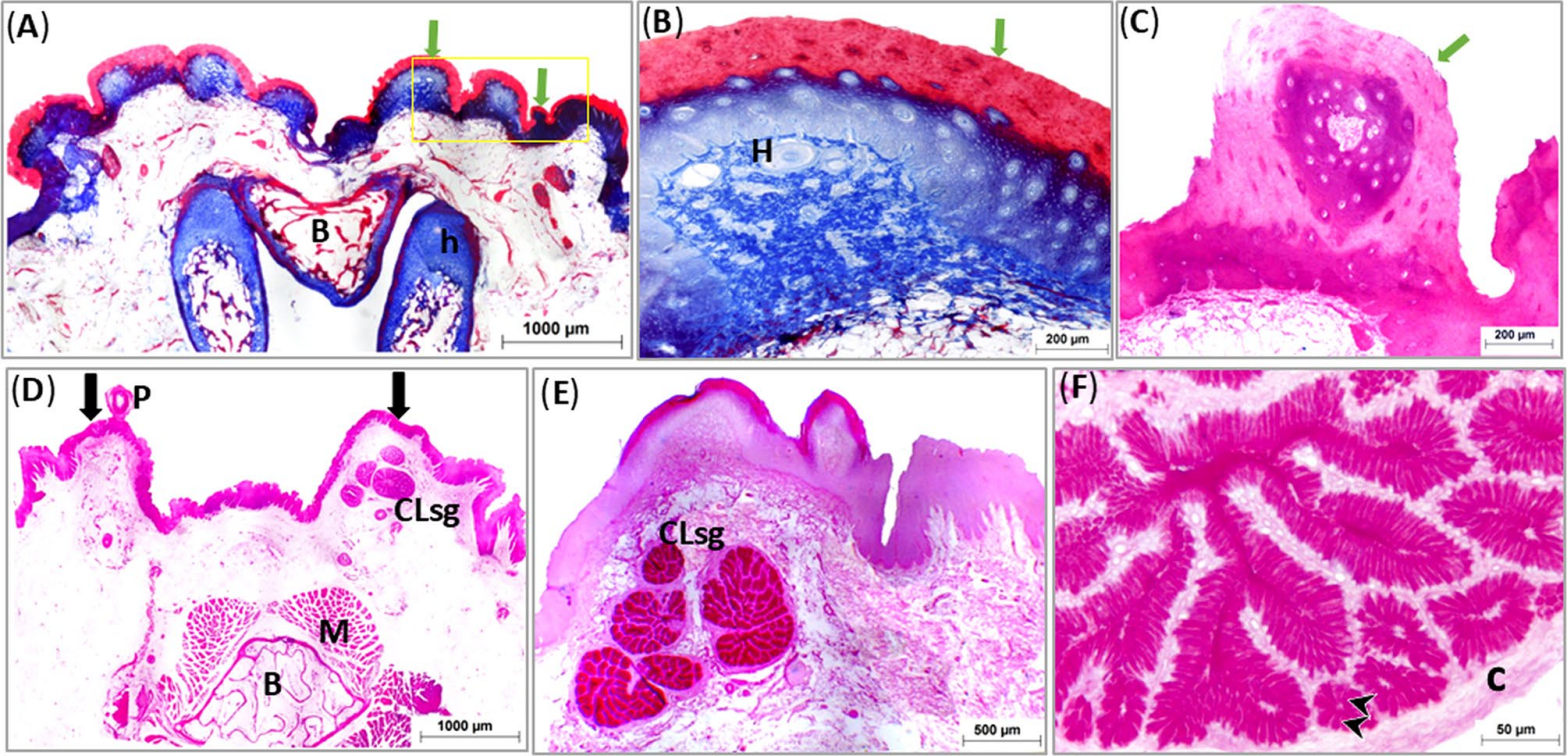
Light micrographs of cross paraffin sections (**A**-**F**) showing structure of the transverse row of lingual papillae (**A**-**C**) and lingual root (**D**-**F**). Photos (**A** & **B**) stained with Masson’s Trichrome, (**C** & **D**) with H&E and (**E** & **F**) with PAS. Note, parakeratinized papillae (green arrow), basihyoid (**B**), two branchial horns (h), Herbst corpuscle (H), caudal lingual gland (CLsg), mucosal elevation (black arrow), muscles (M), mucous secreting cells (black arrow heads) and capsule (**C**)

**Fig. 10:**
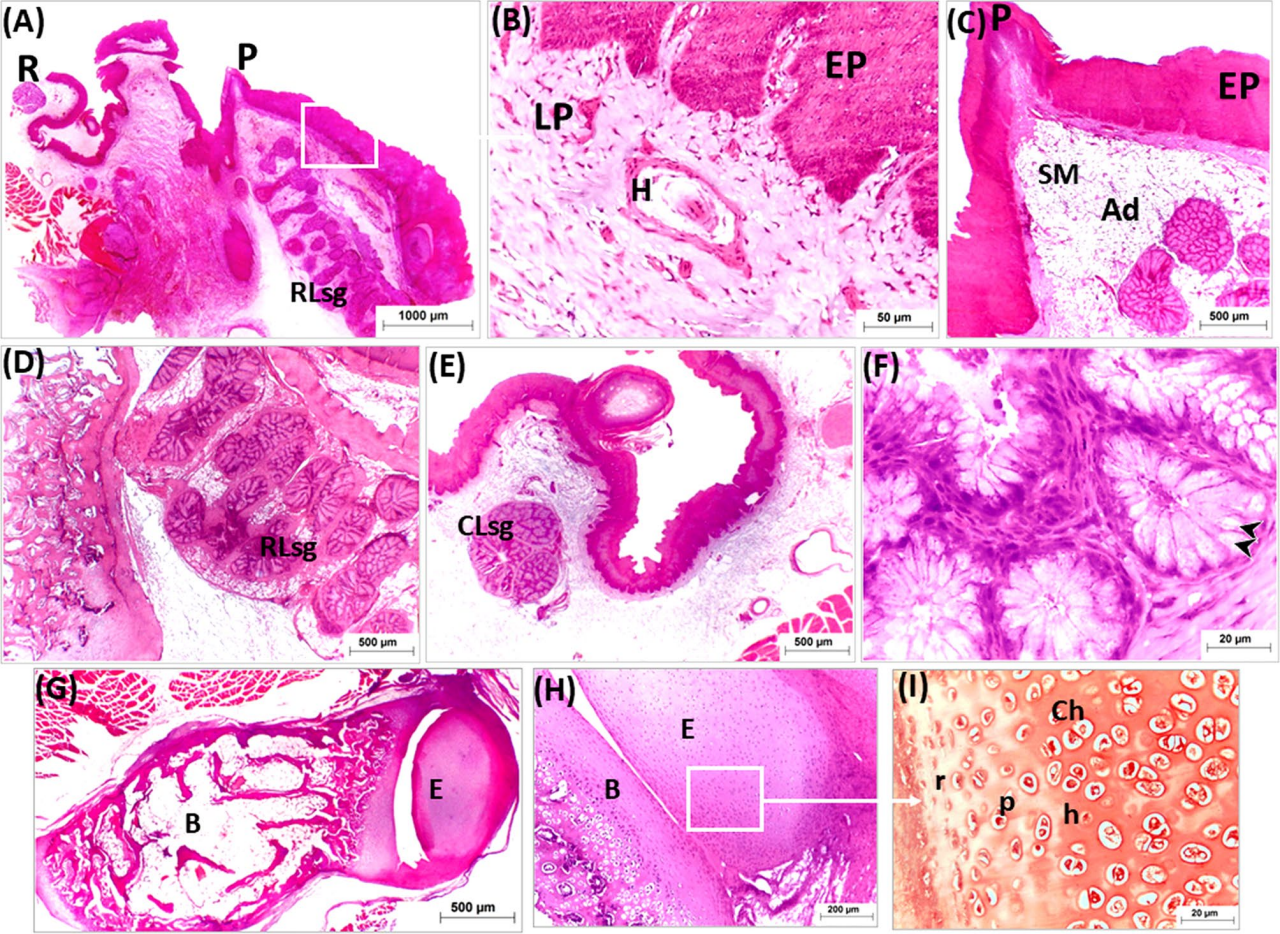
Light micrographs of longitudinal paraffin sections (**A**-**I**) showing structure of the lingual prominence, transverse row of lingual papillae and root. Photos (**A**-**H**) stained with H&E and (**I**) with Safranin O. Note, root (R), papillae (P), epithelium (EP), lamina propria (Lp), Herbst corpuscle (**H**), submucosa (SM), adipose tissue (Ad), rostral lingual salivary gland (RLsg), mucous producing cells (black arrow heads), basihyoid (**B**) entoglossum (**E**), chondrocytes (Ch) resting zone (r), proliferating zone (P) and hypertrophic zones (h)

The lamina propria under the epithelium was constituted of highly vascularized dense irregular connective tissue contained mainly thick bundles of collagenous fibers which stained blue with Masson’s Trichrome (Fig. 5B). The lamina propria was formed of connective tissue papillae interdigitated with epithelial folds from the covering epithelium. The epithelial folding and the papillary interdigitation were more distinct dorsally than ventrally (Fig. 5B, C). In addition, the lamina propria contained several lamellated sensory corpuscles (Herbst corpuscles) and many free nerve endings. The corpuscles were seen through the lamina propria beneath the dorsal epithelium but could not be detected under the ventral epithelium. These corpuscles were accumulated in the area of the lingual nail, rostral to the lingual prominence and also were seen at the base of lingual papillae (Fig. 5C, D. Figures 8C and E, 9B and 10B).

The marginal lingual papillae appeared conical in shape and consisted of core of connective tissue covered by thick para keratinized stratified squamous epithelium. Small conical slightly cornified papillae associated only with the marginal lingual papillae (Figs. 7A, B and E and 8B and D).

The dorsal epithelium of the lingual body formed papillae guard the median lingual groove. At the rostral part of the lingual body, they were small rounded in shape and formed of connective tissue core covered by stratified squamous epithelium (Fig. 7B, D). Caudally they became para keratinized and larger and the largest ones observed just in front the lingual prominence. These papillae were formed of connective tissue core covered by thick para keratinized stratified squamous epithelium (Fig. 8A, C).

The lingual papillae of the transverse row were rounded to conical in shape; they were formed of a core of connective tissue covered by thick para keratinized stratified epithelium (Fig. 9A: C).

The submucosa consisted of a loose connective tissue containing abundant adipose tissue and large blood vessels. The adipose tissue was highly developed especially in the caudal part of lingual body and lingual prominence (Fig. 8A, B).

**Fig. 11:**
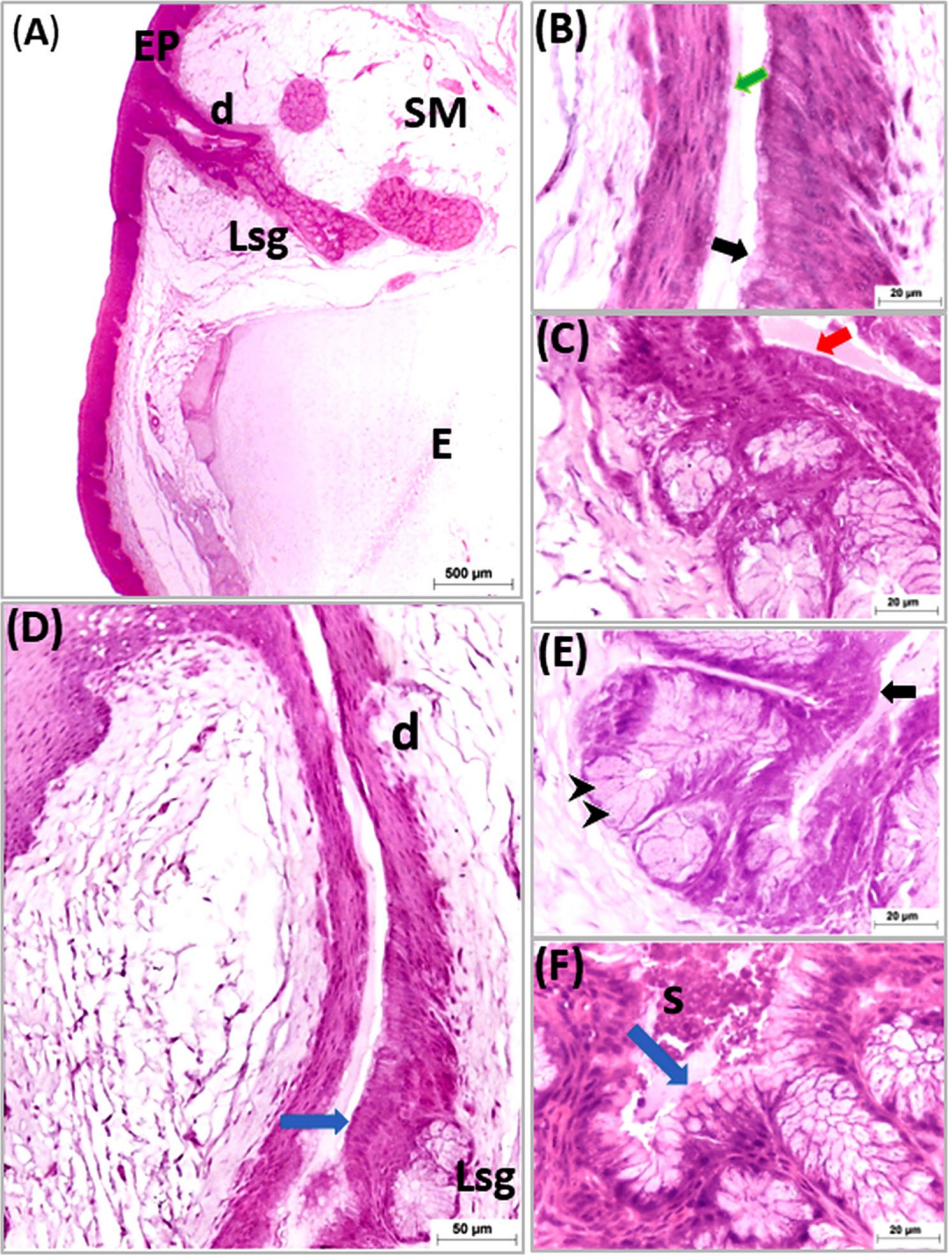
Light micrographs of cross paraffin sections (**A**-**F**) of the lingual salivary glands showing the structure of secretory units and transformation of the ductal epithelium from mucous secreting cells to stratified squamous epithelium. All photos stained with H&E (**A**-**F**). Note, epithelium (EP), submucosa (SM), entoglossum (**E**), lingual salivary glands (Lsg), mucous producing cells (black arrow heads), extralobular duct (d), intralobular duct (blue arrow), stratified columnar epithelium (black arrow stratified cuboidal epithelium. (red arrow), stratified squamous epithelium (green arrow) and secretion (S)

Three aggregations of compound branched mucous tubuloalveolar salivary glands were detected within the submucosa of the tongue surrounded with adipose tissue and blood vessels. The rostral two aggregations of mucous salivary glands of the body and lingual prominence represented the rostral lingual salivary gland, and the third aggregation in the lingual root considered the caudal lingual salivary glands (Figs. 7, 8, 9, 10 and 11).

The stained sections with H&E, showed that the secretory units of both rostral and caudal lingual salivary glands were surrounded with thick capsule which sent connective tissue septa divided them into glandular acini. The units lined with high columnar mucous cells with flattened basally located nuclei, faintly stained foamy cytoplasm and showed intensive reaction for PAS/Alcian blue stains (Figs. 7, 8, 9, 10 and 11).

The duct system of the lingual salivary glands was divided into interlobular and extralobular ducts. The secretory units drained their mucous secretion into a central cavity which was lined by mucous ductal cells. The central cavity drained the secretion into the interlobular duct which was lined by simple columnar epithelia. The interlobular duct was connected to a large extralobular duct which opened through the dorsal and lateral surfaces of the tongue. The extralobular duct was lined by stratified columnar epithelia that gradually transformed to stratified cuboidal which in turn became stratified squamous type which continued with lamina epithelialis of the lingual mucosa (Fig. 11).

The lingual core of geese was formed by the entoglossal bone, situated in the body of tongue, extended rostrally to occupy the entire apex and reached to the rostral free tip of tongue (Figs. 5A, 6, 7A and C and 8A and B). Both ends of the entoglossum were cartilaginous (hyaline type), however the central core was ossified. The caudal end of entoglossal bone terminated in front the transverse row of the lingual papillae whereas it articulated with rostral end of the basibranchial bone forming a synovial joint at the level of transverse row of lingual papillae (Figs. 6 and 10G: I).

The cartilaginous ends of the entoglossal bone had cartilaginous canals which were a sign for bone development occurred before ossification. These canals contained blood vessels and osteogenic cells. Chondrocytes in cartilaginous ends were arranged into discrete zones, resting, proliferating and hypertrophic zones. The central part of the entoglossal bone was formed of cortical compact bone and inner medullary bony trabeculae. The medullary spaces between the bony trabeculae were filled with adipose tissue (Fig. 6).

At the level of the transverse row of the lingual papillae, two branchial horns were observed on both sides of rostral basibranchial bone which were partially ossified (Fig. 9A).

The dorsal mucosal surface of the root of the tongue had two dorsolateral mucosal elevations, carried small cornified conical papillae which formed of core of connective tissue covered by keratinized stratified epithelium. The submucosa contained the caudal lingual glands which aggregated below mucosal elevations and opened at the surface epithelium. The basibranchial bone supported the mucosa and submucosa of the root (Fig. 9A: C).

Bundles of skeletal muscles surrounded the entoglossal bone and basibranchial bones (Figs. 8 and 9).

## Discussion

According to the information we have, this is the first attempt to fully describe the morphology of the geese tongue. Our findings provided clarification on a few specific features of **the geese’s** tongue that adapted to its function. The geese’s tongue was narrow elongated with round thin free apex, that had ventral keratinized lingual nail. Similar observation recorded in domestic goose [2, 3] and the Eurasian collared dove [27, 28], the former author declared that the lingual nail has anterior position and spatula shape, also Jackowiak, Skieresz-Szewczyk [3] added that the characterization of the geese tongue with presence lingual nail may function as a spoon for lifting grains which is a type of feeding mechanism in the Anserinae while Skieresz‐Szewczyk, Prozorowska [2] suggest this function to the rounded apex. However, different observations recorded with multiple researchers, the tongue shape varies according to bird’s species and this variation may related to difference in beak shape, diet and its adapted function [9, 29]. Skieresz‐Szewczyk, Prozorowska [2] declared that the apex shape of the domestic goose varies during the incubation period, it is rounded between 10th and 16th day of incubation, transforms into spatula-like shape on the 17th day of incubation and converts to triangular shape with rounded tip on the 19th day of incubation. However, in pigeon it appears short and triangular and long lanceolate in shape with narrow tapering apex in cattle egret [4]. As well as, a triangular-shaped with a bifurcated tip recorded in the barn owl [5], and elongated flat tongue with rounded apex has multiple acicular processes in the Eurasian common moorhen [9]. While a unique arrowhead-shaped tongue recorded in red-eared slider [30].

Several previous published articles demonstrated that the tongue has two different types of lingual papillae mechanical and gustatory [8, 13, 30–32]. According to the conducted studies, the tongue of Egyptian geese had two groups of mechanical lingual papillae: rostral and caudal. The rostral group was 9–10 narrow spaced small thin conical papillae with pointed apices on the lateral margin of the tongue apex. The caudal group was 6–7 widely spaced large thorn like conical papillae, positioned on the lateral margin of the caudal portion of the lingual apex and the body. With SEM, numerous haired shaped filiform papillae situated between marginal papillae. Presence of these two groups of cornified papillae were necessary reflecting on the geese feeding behavior as geese is grassing birds depend mainly on pasture in their diet with grains as supplement [33]. In accordance with these findings, both sides of the geese’s tongue has four pairs of large conical papillae and nine pairs of small conical papillae, these arrangement is linked functionally with its adaptation for grazing [10], 11 pairs of small conical papillae and 4 pairs of large conical papillae are found on the lateral margines of the lingual body of the domestic geese [3]. The small and large conical papillae are seen also on the lateral lingual sides of duck which is considered as filter-feeder bird depending on grazing and pecking as terrestrial feeding methods [34], but in the rock pigeon, scales-like filiform papillae cover both apex and body making the tongue appear scaly [35] facilatating the easy passage of food particles [36], while in *Anas crecca* and barn owl, these thready papillae are observed on its both lingual lateral sides as food filtration apparatus [5, 11]. In aardvark, abundant conical filiform papillae cover lingual dorsum [32], while in turbot there are submucosal cone shaped papillae that recognized only microscopically [12]. Characterization of bird’s tongue with presence of different types of lingual papillae serve in food collecting, food passage, filtering food particles, food cutting and grazing [3, 10, 34].

The tongue of geese had stratified squamous lining epithelium, keratinization detected dorsally, on ventral free tip of tongue (lingual nail), torus linguae and lingual papillae. There is direct correlation between the lingual keratinization and the feeding behavior of geese. As Keratinization is a sign of tongue modification especially for the herbivorous birds aids in manipulating hard food particles like plant seeds and also for protecting lingual structure against any hard foreign material [3, 37]. Similar findings recorded by Jackowiak, Skieresz-Szewczyk [3]. Abumandour [36] also reported that the lingual dorsum of house sparrow is keratinized. However the barn owl, keratinization is detected ventrally and over the caudal part of the tongue [5] and gradually disappears caudally to be absent in the root of both White-Eared Bulbul and Bronze Fallow Cockatiel [38]. While different degree of keratinization is recorded in the tongue of Polyborus plancus [39]. According to mammals species different observations are recorded as in Gracilinanus microtarsus, where the keratinization is observed only on the ventral lingual epithelium [7], and caudolateral to the lingual torus in roe deer [8]. But for reptile species; Heremites vittatus, the keratinization appears in the fore tongue, absent in the mid- and hind tongue of [14]. While, no sign of keratinization is observed in the tongue of turtles [40]. As well as Several goblet cells are distinguished among the surface squamous stratified epithelial cells in the tongue of turbot [12].

Many researchers demonstrated that the tongue has gustatory function through distinguish taste buds and or gustatory papillae in various species of birds, fish, reptiles and animals as, domestic goose [3], the Eurasian collared dove [27], turbot [12], red-eared slider [30], the Egyptian buffalo [41], and Gracilinanus microtarsus [7]. Although neither taste buds nor gustatory papillae had been distinguished in this investigation, which are the main structures responsible for gustation, the geese tongue had special sensory corpuscles (Herbest corpuscles) which were responsible for food recognition, were spread all over the tongue accumulated in the lingual nail, rostral to the lingual prominence and at the base of lingual papillae. Similar findings reported by Jackowiak, Skieresz-Szewczyk [3] in domestic goose shown that heavily accumulation of Herbst corpuscles in the lingual nail and lingual prominence are necessary for food recognition. Different researchers studied these sensory structures as Crole, du Plessis [42] in the oropharynx of ostrich and emu which consider them as mechanoreceptors, Crole and Soley [43] also considers the emu tongue a sensory organ for taste and touch due to presence taste receptors and Herbst corpuscles, and accumulation of these Herbst corpuscles in the median palatine and ventral ridges in the ostrich oropharynx denote these structures as sensory organs aid in food handling and transportation [44]. Madkour, Choudhary [45], recognize them near glandular lobe of preen gland of dove, as well as Soliman and Madkour [46] that Herbest corpuscles with various types of corpuscles as Grandry, Merkel and Ruffini are detected in the duck and quail beak serving as specific receptors respond to vibration, pressure and stretching sensation.

In the current study, three aggregations of compound branched mucous tubuloalveolar salivary glands were detected within the submucosa, consisting of columnar mucous cells arranged as glandular acini showed intensive reaction for PAS and Alcian blue stains that strong reaction. Similar observations recorded in glands of lingual apex and body of Rock Pigeon, but the root glands react negatively for PAS during young and adult ages [47] and lingual glands of the Egyptian Endemic Bridled Skink [14]. Different observations recorded by different researchers as; in the Eurasian collared dove by El-Mansi, El-Bealy [27] where the units of salivary glands exhibit intense alcianophilic reactions. While in the Gracilinanus microtarsus, the lingual salivary glands have three types of acini; serous, mucous and mixed type [7]. Both mixed and serous glands are recognized in the tongue of Sulawesi bear, but the secretion composition differs between young and adult ages [48]. These differences in the composition of the glandular secretion reflect the species adaptation to their specific diets [49]. Wassif and El-Hawary [50] suggested that the lingual.

gland’s secretion acts as a lubricant material on the lingual dorsum facilitating the movement of the food particles, transportation, and swallowing.

## Conclusions

The microscopic structure of the geese tongue was described for the first time in this investigation revealed that the tongue had several specialized structures as lingual nail, core of entoglossal bone that articulated with basibranchial bone, and two groups of marginal cornified papillae. As well as there were specialized sensory corpuscles (Herbest corpuscles) scattered along the whole tongue beneath the dorsal epithelium thought to be responsible for gustation. Aswell as, these findings advance our knowledge of the gross and microscopic anatomy of the tongue in geese and help to enhance the productivity of Aves.

## Data Availability

Requests for data can be made to the corresponding author.
